# Uniportal video-assisted thoracoscopic surgery without drainage-tube placement for pulmonary wedge resection: a single-center retrospective study

**DOI:** 10.1186/s13019-022-02053-9

**Published:** 2022-12-17

**Authors:** Seha Ahn, Youngkyu Moon

**Affiliations:** Department of Thoracic and Cardiovascular Surgery, Eunpyeong St. Mary’s Hospital, College of Medicine, The Catholic University of Korea, 1021, Tongil-ro, Eunpyeong-gu, Seoul, 03312 Republic of Korea

**Keywords:** Uniportal VATS, Pulmonary wedge resections, No drainage tube placement, Residual pneumothorax, Partial pleural adhesions

## Abstract

**Background:**

Uniportal video-assisted thoracoscopic surgery without drainage-tube placement has been demonstrated to be safe and feasible for select situations. The purpose of this study is to assess the demographic, baseline, and intraoperative characteristics of patients who developed residual pneumothorax after thoracic surgery without drainage-tube placement.

**Methods:**

We reviewed the records of all patients who underwent pulmonary wedge resection via uniportal video-assisted thoracoscopic surgery without drainage-tube placement between May 2019 and May 2022. The decision to omit chest-tube drainage was originally made on a case-by-case basis, using internal criteria. Postoperative chest radiography was performed on the day of surgery, on postoperative day 1, at the first outpatient visit, and at 1 month after surgery.

**Results:**

A total of 134 patients met the selection criteria; 23 (17.2%) had residual pneumothorax on chest radiography on postoperative day 1, and 5 (3.7%) had residual pneumothorax at the first outpatient visit. Only 1 patient (0.7%) had residual pneumothorax on chest radiography at 1 month after surgery; this patient did not require chest-tube insertion or any other intervention. The presence of partial pleural adhesions independently increased the risk for postoperative residual pneumothorax on chest radiography, whereas older patient age reduced the risk.

**Conclusions:**

Uniportal video-assisted thoracoscopic surgery for pulmonary wedge resection without drainage-tube placement is both safe and feasible for carefully selected patients. Most patients with residual pneumothorax in our study experienced spontaneous resolution, and none required reintervention.

## Introduction

Over the last few decades, video-assisted thoracoscopic surgery (VATS) with single-lung ventilation has become a well-established modality for pulmonary resection [1]. Modifications to the conventional approach to VATS have been made to reduce surgical stress, including reducing the number of access ports, avoiding the use of an endotracheal tube during surgery, and avoiding chest-tube drainage after VATS pulmonary wedge resection [2–4]. Some surgeons have adopted uniportal (single-port) VATS pulmonary resection as an alternative to multiport VATS [5–7]. Pompeo et al. reported their experience with conventional VATS using intravenous anesthesia without endotracheal intubation (tubeless VATS) for pulmonary nodule resection in 2004 [4]. In the same year, Watanabe et al. reported their experience with skipping chest-tube placement after VATS for pulmonary wedge resection [3]. Recently, tubeless uniportal VATS without drainage-tube placement has been introduced and demonstrated to be safe and feasible for selected patients [8–10].

Without a chest tube present for drainage after VATS for pulmonary wedge resection, there is a risk for residual pneumothorax or pleural effusion requiring reintervention during the index hospital stay [3, 11–13]. We therefore aimed to assess the demographic, baseline, and intraoperative factors associated with development of residual pneumothorax after uniportal VATS for pulmonary wedge resection without drainage-tube placement.

## Material and methods

### Patient population

We conducted a single-center, retrospective analysis of 461 patients who underwent uniportal VATS by a single surgeon between May 2019 and May 2022. The decision to omit chest-tube drainage for each patient was made at the time of surgery, based on the following criteria: a peripheral pulmonary lesion ≤ 2 cm in size, the absence of severe pleural adhesions on intraoperative inspection, ≤ 3 individual unilateral wedge resections, and the absence of air leaks confirmed through a digital drainage system (DDS). Patients with bleeding disorders, currently taking an anticoagulants and antiplatelet drugs, and patients requiring anatomical pulmonary resection such as segmentectomy or lobectomy were excluded.

### Surgical technique

**Fig. 1:**
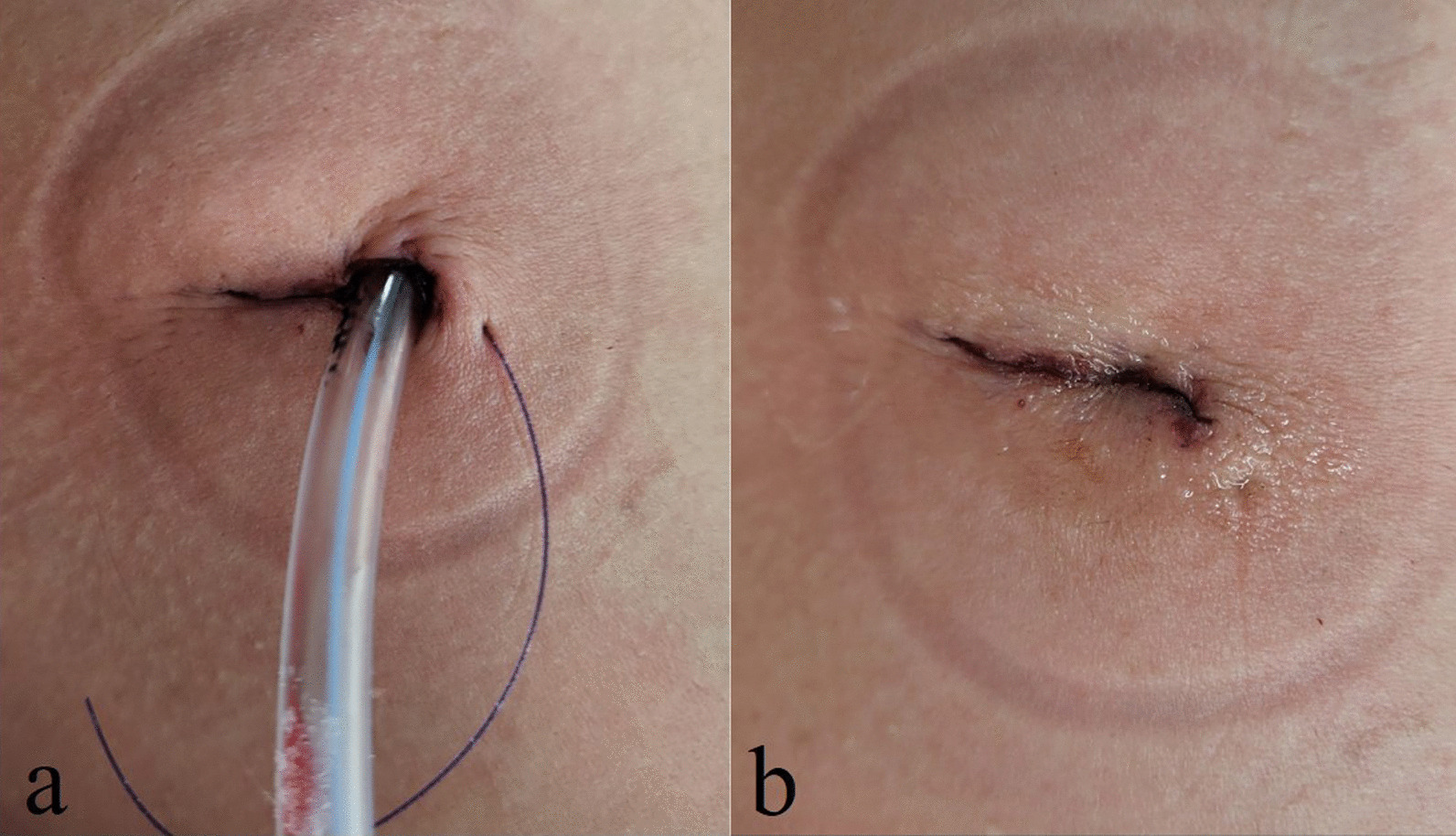
Closed working incision with a 20-French chest tube after uniportal VATS pulmonary wedge resection (**a**). Closed working incision was covered with topical tissue adhesive after cutting the remainder of secured thread (**b**)

All procedures were performed with patients in the lateral decubitus position. The surgical incision, approximately 2 cm in length, was made in the anterior axillary line at either the fourth or fifth intercostal space (ICS), depending on the location of the target lesion. For both upper lobes and the right middle lobe, the typical working incision was made at the fourth ICS. For both lower lobes, the working incision was made at the fifth ICS. The working port was covered with a small wound protector (W-SHIELD RETRACTOR X-S; SNT MEDICAL, Seoul, Korea). A 5-mm, 30° scope was positioned at the upper side of the incision by the surgical assistant. A curved suction tip, grasping instruments, and articulating endostaplers were inserted through the single incision. In patients with malignancy (primary lung cancer or lung metastasis), the resection margin for the pulmonary wedge resection was 1 cm, or the size of the nodule. None of the patients used polyglycolic acid (PGA) sheets or fibrin adhesives to reinforce staple lines after pulmonary wedge resection. All patients received an intercostal nerve block using 1 mL of bupivacaine in each space beneath the lower margin of the third through seventh ribs. Before closing the incision, a 20-French chest tube was inserted through the lower part of the incision. The working incision was closed in layers, and the skin was closed using a unidirectional absorbable barbed suture (V-Loc 180®; Medtronic, Mansfield, MA) with leaving a thread (Fig. 1a).

### Air leak test

The chest tube was connected to a DDS (Thopaz; Medela Healthcare, Baar, Switzerland) with a suction pressure of -15 cm H_2_0, and the patient was moved from the lateral decubitus position to a supine position. If an air leak turns out to an airflow greater than 0 mL/min in the DDS, the chest tube was not planned to be removed. On the other hand, if airflow was confirmed as 0 mL/min by DDS, the anesthesiologist provided an artificial deep inspiration using a bag-valve mask while the chest tube was removed. The secured thread was then pulled forward to tighten the working-incision closure. Finally, the closed working incision was covered with topical tissue adhesive (Indermil® flexifuze™; Connexicon Medical Ltd., Tallaght, Republic of Ireland) after cutting the remainder of secured tread (Fig. 1b).

### Postoperative management

Erect anteroposterior chest radiography (CXR) was performed for all patients in the recovery room, 20 min after completing the procedure. Posteroanterior CXR was performed in the morning of postoperative day 1 (POD 1). Subcutaneous emphysema was defined as a detectable radiolucent area in the soft tissue extending from the surgical wound. Any patients with symptoms consistent with either pleural effusion or pneumothorax in whom the area of air occupied more than 25% of the pleural space underwent thoracentesis or chest-tube drainage. A residual pneumothorax was defined as a radiologically detectable apical pleural space. A residual pleural effusion was defined as blunting of the costophrenic angle on the side of the procedure. After hospital discharge, all patients underwent postoperative CXR at the first outpatient visit and at 1 month after surgery.

### Statistical analysis

Continuous variables were expressed as the mean ± standard deviation, and categorical variables were presented as the number and frequency (%). A logistic regression model was used to evaluate the factors associated with residual pneumothorax on CXR. All statistical analyses were performed using R software (RStudio version 4.2.0; https://www.r-project.org/). A *P* value of less than 0.05 was regarded as statistically significant.

## Results

A total of 134 patients underwent pulmonary wedge resection via uniportal VATS without drainage-tube placement. The patient population included 53 men and 81 women, with a mean age of 45 years (range 13–83 yr). The indication for surgery was benign lung disease in 28 patients (20.9%), malignant lung disease in 86 (64.2%), and pneumothorax in 20 (14.9%). The other demographic and baseline characteristics are presented in Table 1. A total of 20 patients (14.9%) had partial pleural adhesions visible on intraoperative inspection; adhesions were absent in 114 patients (85.1%). The number of staple cartilages used was grouped into few (1–2), some (3–5), and many (6–8). The average operative time was 40.1 ± 17.6 min (Table 2).

**Table 1:** Patient’s demographics and baseline characteristics (n = 134)

Variable	N(%) or mean (± SD)
Age, y	44.7 ± 15.2
Sex	
Male	53 (39.6%)
Female	81 (60.4%)
Body mass index, kg/m^2^	23.0 ± 3.8
Current or former smoker	30 (22.4%)
Pulmonary function	
FEV1 (%)	106.1 ± 15.2
DLCO (%)	95.8 ± 16.4
Affected lobe	
Right upper	29 (21.6%)
Right middle	13 (9.7%)
Right lower	30 (22.4%)
Left upper	19 (14.2%)
Left lower	22 (16.4%)
More than one lobe	21 (15.7%)
Diagnosis	
Benign lung disease	28 (20.9%)
Malignant lung disease	86 (64.2%)
Pneumothorax	20 (14.9%)
Comorbidity	
Non-lung diseases	127 (94.8%)
Lung diseases (COPD, Asthma, ILD)	7 (5.2%)

*SD* standard deviation, *FEV1* forced expiratory volume in 1 s, *DLCO* diffusing capacity of the lung for carbon monoxide, *COPD* chronic obstructive pulmonary disease, *ILD* interstitial lung disease

**Table 2:** Operative results (n = 134)

Variable	N(%) or mean (± SD)
Adhesion	
None	114 (85.1%)
Partial	20 (14.9%)
Number of staple cartilage	
Few (1–2)	29 (21.6%)
Some (3–5)	90 (67.2%)
Many (6–8)	15 (11.2%)
Operation duration, min	40.1 ± 17.6

*SD* standard deviation

Of the 134 patients, 19 (14.2%) developed postoperative subcutaneous emphysema on POD 1. Residual pneumothorax was present in 23 patients (17.2%) on POD 1, in 5 patients (3.7%) at the first outpatient visit, and in only 1 patient (0.7%) at 1 month. Two patients (1.5%) had a residual pleural effusion on POD 1, which resolved by the first outpatient visit. The mean duration of postoperative stay was 3.8 days. No patients required reintervention (thoracentesis or chest tube drainage) because the area of air occupied less than 20% of the pleural space during the index hospital stay, and no patients required readmission; there was no mortality (Table 3).

**Table 3:** Treatment outcomes (n = 134)

Variable	N(%) or mean (± SD)
Adverse events	
Subcutaneous emphysema	19 (14.2%)
Reintervention during hospital stays	0 (0%)
Residual pneumothorax on Postoperative CxR	
On POD 1	23 (17.2%)
At first outpatient visit	5 (3.7%)
At 1 month	1 (0.7%)
Residual pleural effusion on Postoperative CxR	
On POD 1	2 (1.5%)
At first outpatient visit	0 (0%)
At 1 month	0 (0%)
Duration of postoperative stay, d	3.8 ± 2.5
Readmission	0 (0%)
Mortality	0 (0%)

*SD* standard deviation, *CxR* chest radiography, *POD* postoperative day

The demographic and baseline characteristics of the 23 patients with residual pneumothorax on POD 1 are shown in Table 4. There were 13 men and 10 women experiencing this complication, with a mean age of 33 years (range 13–56 yr) and a mean body mass index of 21.0 kg/m^2^ (range 14.7–32.3 kg/m^2^). Seven patients were current or former smokers. Approximately half of the patients (47.8%) were diagnosed with pneumothorax. Seven of the patients with POD1 residual pneumothorax (30.4%) had partial pleural adhesions visible at the time of surgery (Table 4).

**Table 4:** Patients with residual pneumothorax on Postoperative CxR: Demographics and Baseline Characteristics (n = 23)

Variable	N(%) or mean (± SD)
Age, y	32.6 ± 12.3
Sex	
Male	13 (56.5%)
Female	10 (43.5%)
Body mass index, kg/m^2^	21.0 ± 4.3
Current or former smoker	7 (30.4%)
Affected lobe	
Single lobe	21 (91.3%)
More than one lobe	2 (8.7%)
Diagnosis	
Benign lung disease	3 (13.0%)
Malignant lung disease	9 (39.1%)
Pneumothorax	11 (47.8%)
Comorbidity	
Non-lung diseases	22 (95.7%)
Lung diseases (COPD, Asthma, ILD)	1 (4.3%)
Adhesion	
None	16 (69.6%)
Partial	7 (30.4%)
Number of staple cartilage	
Few (1–2)	6 (26.1%)
Some (3–5)	15 (65.2%)
Many (6–8)	2 (8.7%)
Operation duration, min	36.5 ± 11.8

*SD* standard deviation, *COPD* chronic obstructive pulmonary disease, *ILD* interstitial lung disease

Univariable analysis showed that a preoperative diagnosis of pneumothorax (odds ratio [OR], 10.20; *P* = 0.002) and the presence of partial pleural adhesions (OR 3.30; *P* = 0.027) were associated with an increased risk of postoperative residual pneumothorax. In contrast, older patient age (OR 0.93; *P* < 0.001) and higher body mass index (OR 0.81; *P* = 0.006) were associated with a lower risk for postoperative residual pneumothorax. Multivariable analysis confirmed that the presence of partial pleural adhesions (OR 8.57; *P* = 0.004) was associated with an increased risk for postoperative residual pneumothorax, whereas older patient age (OR 0.94; *P* = 0.021) was associated with a reduced risk (Table 5).

**Table 5:** Regression analysis of the risk factors for residual pneumothorax

Variable	Univariable regression analysis		
	OR	95% CI	*P* value
Age, years	0. 93	0.89–0.96	< 0.001
Gender			
Male	Reference		0.072
Female	0.43	0.17–1.08	
BMI	0.81	0.70–0.94	0.006
Smoking	1.67	0.62–4.55	0.313
Affected lobe			
Single lobe	Reference		0.322
More than one lobe	0.46	0.10–2.13	
Diagnosis			
Benign lung tumor	Reference		
Malignant lung tumor	0.97	0.24–3.88	0.970
Pneumothorax	10.20	2.30–45.00	0.002
Comorbidity			
None	Reference		0.836
Lung diseases	0.80	0.09–6.94	
Adhesion			
None	Reference		0.027
Partial	3.30	1.14–9.52	
Number of staplers			
Few (1–2)	Reference		
Some (3–5)	0.77	0.27–2.20	0.622
Many (6–8)	0.59	0.10–3.36	0.552
Operation duration, min	0.98	0.95–1.01	0.283

Variable	Multivariable regression analysis		
	OR	95% CI	*P* value
Age, years	0.94	0.88–0.99	0.021
Gender			
Male	Reference		0.699
Female	0.79	0.24–2.60	
BMI	0.97	0.82–1.14	0.673
Diagnosis			
Benign lung tumor	Reference		
Malignant lung tumor	1.40	0.29–6.74	0.672
Pneumothorax	2.93	0.32–26.80	0.341
Adhesion			
None	Reference		
Partial	8.57	1.96–37.40	0.004

*CI* confidence interval, *OR* odds ratio, *BMI* Body mass index

## Discussion

Our promising experience with uniportal VATS pulmonary wedge resection without drainage-tube placement indicates that this approach is both safe and feasible: postoperative CXR revealed residual pneumothorax in 23 patients on POD 1, in 5 patients at the first outpatient visit, and in only a single patient at 1 month. We consider this rate of residual pneumothorax acceptable because all resolved spontaneously; none required reintervention. However, our multivariable analysis reveals factors related to residual pneumothorax on CXR that surgeons should be aware of: the presence of partial pleural adhesions and younger patient age were associated with this complication. Surgeons should be more cautious removing chest-tube drainage at the end of surgery in such patients.

Since Watanabe et al. reported their successful avoidance of chest-tube placement after VATS for pulmonary wedge resection, many surgeons have established their own inclusion and exclusion criteria for omitting chest drainage [8, 9, 11, 13–19]. Huang and colleagues recently published a systematic review and meta-analysis of the efficacy and safety of omitting chest drains after VATS based on 10 studies (4 randomized controlled trials [RCTs] and 6 non-RCTs). The patient selection in these studies varied slightly in baseline patient characteristics and the methods used for testing intraoperative air leakage [10].

Our inclusion criteria included lesion size and location (peripheral pulmonary lesions of ≤ 2 cm), the absence of severe pleural adhesions on intraoperative inspection, and ≤ 3 individual unilateral wedge resections. We excluded from our review patients prone to bleeding and those requiring anatomic pulmonary resection. Our criteria are similar to those used by Liu and colleagues for patient selection, except Liu excluded patients with parenchymal lung disease [8]. Our multivariable logistic regression analysis showed that the presence of partial pleural adhesions increases the risk for postoperative residual pneumothorax. Although we excluded patients with severe pleural adhesions from consideration for this procedure modification, there appears to be unrecognized lung damage caused by partial pleural adhesions [3].

Liu and colleagues reported that a preoperative diagnosis of spontaneous pneumothorax increases the risk for an abnormal postoperative CXR (OR 7.44; *P* < 0.001 on univariable analysis; OR, 5.747; *P* = 0.001 on multivariable analysis) [8]. We found that this risk was statistically significant on univariable analysis but not multivariable analysis. As seen in Table 4, approximately half of our patients were diagnosed with preoperative pneumothorax. It is possible that preoperative pneumothorax was not a statistically significant predictor of residual postoperative pneumothorax on multivariable analysis because only 20 patients (14.9%) had preoperative pneumothorax. Because this affected only a small percentage of patients preoperatively, older age was associated with a reduced risk for postoperative residual pneumothorax on multivariable analysis (Table 5).

The review by Huang and colleagues also assessed the method of performing the intraoperative air leakage test. This test can be done in a variety of ways, including the water sealing method, thoracoscopic inspection, use of a vacuum ball, and digital measurement [10]. In our method, no water sealing method was performed to check for air leaks when performing pulmonary wedge resections. Instead, we prefer using a DDS, which allows us to precisely time chest-tube removal and also provides constant negative pleural pressure [8, 20, 21]. Airflow in all patients was confirmed as 0 mL/min by DDS after position change, and all chest tubes were removed in the operating room.

Many studies have shown that the length of stay is 1.53 days shorter for individuals with no chest tube than for individuals with a chest tube [10]. One of our strategies to shorten hospital stays is to apply tissue adhesive to the VATS incision. Patients are able to shower on POD 1 and a dressing does not need to be reapplied. Of our 143 patients, 82 (61%) were discharged before POD 3. Our mean postoperative stay of 3.8 days (Table 3) was skewed by the fact that more than half of our patients had malignant lung disease, either early lung cancer or lung metastasis from another primary; many of these patients wanted to stay in the hospital until their final pathologic report was ready because of the distance from their home to the hospital.

## Limitations

There are several limitations to our study. First, the number of patients was small because all procedures were performed by a single surgeon at a single institution. Second, our study was nonrandomized and retrospective, and our decision algorithm to omit chest-tube placement could have introduced selection bias. Lastly, we believe that the postoperative stay can be shortened by strengthening our selection criteria for uniportal VATS pulmonary wedge resection without drainage-tube placement.

## Conclusion

Pulmonary wedge resection via uniportal VATS without drainage-tube placement is both safe and feasible for carefully selected patients. Although CXR revealed residual pneumothorax in 23 patients (17.2%) on POD 1, most patients underwent spontaneous resolution and none required reintervention. Older age was associated with a reduced risk for postoperative residual pneumothorax on our multivariable analysis, whereas the presence of partial pleural adhesions independently increases the risk for postoperative residual pneumothorax. These factors should be considered when considering the omission of chest-tube drainage after uniportal VATS pulmonary wedge resection.

## Data Availability

The datasets used and/or analyzed during the current study are available from the corresponding author on reasonable request.

## Abbreviations

VATS: Video-assisted thoracoscopic surgery
DDS: Digital drainage system
ICS: Intercostal space
PGA: Polyglycolic acid
CXR: Chest radiography
POD: Postoperative day
